# Quality of reporting and risk of bias: a review of randomised trials in occupational health

**DOI:** 10.1136/oemed-2020-107038

**Published:** 2021-06-23

**Authors:** Christina Tikka, Jos Verbeek, Sharea Ijaz, Jan L Hoving, Julitta Boschman, Carel Hulshof, Angela G de Boer

**Affiliations:** 1 Department of Public and Occupational Health, Coronel Institute of Occupational Health, Amsterdam Public Health Research Institute, Amsterdam UMC Location AMC, Amsterdam, The Netherlands; 2 Occupational health department, Finnish Institute of Occupational Health (FIOH), Kuopio Regional Office, Kuopio, Finland; 3 Department of Public and Occupational Health, Coronel Institute of Occupational Health, Cochrane Work Review Group, Amsterdam UMC Location AMC, Amsterdam, The Netherlands; 4 NIHR ARC West, University of Bristol Medical School, University of Bristol, Bristol, UK

**Keywords:** clinical trial, occupational health, public health

## Abstract

**Objectives:**

To assess the reporting quality of randomisation and allocation methods in occupational health and safety (OHS) trials in relation to Consolidated Standards of Reporting Trials (CONSORT) requirements of journals, risk of bias (RoB) and publication year.

**Methods:**

We systematically searched for randomised controlled trials (RCTs) in PubMed between 2010 and May 2019 in 18 OHS journals. We measured reporting quality as percentage compliance with the CONSORT 2010 checklist (items 8–10) and RoB with the ROB V.2.0 tool (first domain). We tested the mean difference (MD) in % in reporting quality between CONSORT-requiring and non-requiring journals, trials with low, some concern and high RoB and publications before and after 2015.

**Results:**

In 135 articles reporting on 129 RCTs, average reporting quality was at 37.4% compliance (95% CI 31.9% to 43.0%), with 10% of articles reaching 100% compliance. Reporting quality was significantly better in CONSORT-requiring journals than non-requiring journals (MD 31.0% (95% CI 21.4% to 40.7%)), for studies at low RoB than high RoB (MD 33.1% (95% CI 16.1% to 50.2%)) and with RoB of some concern (MD 39.8% (95% CI 30.0% to 49.7%)). Reporting quality did not improve over time (MD −5.7% (95% CI −16.8% to 5.4%).

**Conclusions:**

Articles in CONSORT-requiring journals and of low RoB studies show better reporting quality. Low reporting quality is linked to unclear RoB judgements (some concern). Reporting quality did not improve over the last 10 years and CONSORT is insufficiently implemented. Concerted efforts by editors and authors are needed to improve CONSORT implementation.

**Key messages**

**What is already known about this subject?**

- The hallmark of randomised controlled trials is the random allocation of study participants but it has since long been recognised that randomisation and allocation methods are not well reported in general medical journals.

**What are the new findings?**

- In 135 articles of occupational health intervention studies, on average authors reported sufficiently on only 37.4% (95% CI 31.9% to 43.0%) of the required Consolidated Standards of Reporting Trials (CONSORT) checklist items regarding randomisation and allocation concealment, with only 10% of articles reaching 100%.
- In journals that require authors to use CONSORT reporting guidelines, reporting quality was 31 percentage points better (95% CI 21.4% to 40.7%) than in non-requiring journals.
- The average reporting quality of studies with low risk of bias was 33.1 percentage points better (95% CI 16.1% to 50.2%) than of studies with a high risk of bias.
- Over the past 10 years, the average reporting quality of randomisation and allocation concealment methods in occupational health and safety (OHS) trials did not improve.

**How might this impact on policy or clinical practice in the foreseeable future?**

- Findings demonstrate the need for journal editors and peer-reviewers to improve CONSORT adherence in randomised OHS trial reports.
- Trial authors need to improve reporting of sequence generation, allocation concealment and randomisation implementation methods in trial reports.

## Introduction

As researchers, we have an implicit sense that some studies are better than others. To this end, we judge how studies are set up and whether a risk exists that the results are biased.^1 2^ For this, proper reporting of how research data was collected, analysed, and interpreted is crucial regardless of the study type. Already twenty years ago, researchers called for better reporting of studies, which resulted in reporting guidelines for almost any study type.^3^

Even though randomised controlled trials (RCTs) are considered the most rigorous scientific method to evaluate the effectiveness of interventions,^4^ evaluating the risk of bias (RoB) is indispensable to judge if findings can be trusted. When randomisation was not carried out properly or the allocation of participants was not maintained throughout the study, the results may be biased leading to false positive or false negative findings.^5–7^ Yet, missing information in study reports may make it difficult or impossible to judge the RoB and the truthfulness of the results.

To ensure that methods are well documented and to improve the reporting quality of RCTs, the Consolidated Standards of Reporting Trials (CONSORT) has been developed. It is a list of items to be reported in RCTs ‘for which there was evidence, whenever possible that not adequately reporting this information could lead to biased estimates of the benefits of the intervention under investigation’.^8^ According to the CONSORT statement, articles reporting RCTs should include specific information about, among others, the randomisation methods and allocation procedure.^9^ The CONSORT checklist addresses the minimum set of items deemed fundamental to be reported in any randomised trial.^8^ It has been developed for RCTs with an extension for cluster RCTs (cRCTs), which are conducted in many health fields including occupational health and safety (OHS). From the reported information, the reader can judge if allocation was truly random and was maintained throughout the study and assess the RoB for these items.^10^ In 2010, the CONSORT statement was revised and published in ten journals. Since then the updated statement has been endorsed by some journals and not by others. One can assume that more attention is drawn to the statement over the years, but it is unclear whether compliance with CONSORT has improved over time. Compliance with CONSORT has been studied in many medical fields with the general conclusion that reporting quality needs to be improved.^11–17^ However, compliance with CONSORT of trials reported in OHS journals and the association to the RoB in studies has not been evaluated.

### Objective

The aim of this study is to assess the reporting quality of randomisation and allocation methods of trials published in OHS journals and to evaluate if the reporting quality differs between earlier and more recent years of publication, CONSORT-requiring and non-requiring journals, and studies with high, some concern and low RoB.

## Methods

We defined reporting quality as percentage compliance with CONSORT checklist items in articles, with 100% indicating full compliance (highest reporting quality) and 0% implying no CONSORT item being reported (lowest reporting quality). We calculated the minimum required sample size to be 100 articles to detect a prevalence of 3% reporting quality different from zero with a power of 0.8 and p being 0.05.^18^ We conducted a systematic literature search in 18 scientific OHS journals using MEDLINE via PubMed. We searched for RCTs published between 1 January 2010 (the year the CONSORT statement was revised)^9^ and 28 May 2019. The full search strategy is provided in online supplemental table a.

10.1136/oemed-2020-107038.supp1 Supplementary data

We included RCTs with workers or workplaces as participants, irrespective of the type of intervention and comparison. We excluded cross-over trials, protocols, pilot studies, exposure studies, and studies that only reported secondary outcomes or cost-effectiveness analysis results because of more complicated reporting issues. We retrieved full-text articles of included studies to extract data on the year of publication, journal, randomisation methods and allocation process. We classified journals as CONSORT-requiring or non-requiring journals, based on the information provided on the journals’ websites. We defined a journal as CONSORT-requiring, if authors are required to comply with the CONSORT statement or checklist^9 19^ when submitting an article. We distinguished between RCTs and cRCTs and classified articles with missing information on the type of randomisation method as RCTs.

We used the CONSORT 2010 checklist^9^ and the extension for cRCTs^19^ to measure the reporting quality of the randomisation method and allocation process in articles. Assessment was done by one and checked by a second author. Disagreements were resolved via discussion. The checklist is structured into a fixed set of items that need to be included in a journal article when reporting a randomised trial. We applied the checklist items required for reporting the sequence generation, allocation concealment, randomisation implementation method and type. Those are four items for RCTs (8a, 8b, 9 and 10) and eight items for cRCTs (8a, 8b, extension 8b, 9, extension 9, 10a, 10b, 10c) (online supplemental table b). We calculated the reporting quality as percentage score (0%–100%) using the number of items reported divided by the number of items required according to CONSORT. For each article, data extraction and assessment of reporting quality was done by one and checked by a second author. We calculated the mean and SD of the reporting quality score per journal, journal type (CONSORT requirement yes or no), publication year (2010–2014 and 2015–2019), study type (RCTs and cRCTs) and RoB (low, some concern and high).

The Cochrane risk of bias tool (ROB V.2.0) is structured into five domains of signalling question. Each domain focuses on different aspects of the trial. Based on the answers to the signalling questions, the judgement of the RoB can be generated by an algorithm as either ‘low RoB’, ‘some concern’ or ‘high RoB’. We used the first domain of ROB V.2.0 and the proposed algorithm to assess the RoB arising from the randomisation process (risk of selection bias) for each included trial.^10^ We used study IDs to identify articles that report on the same study and agreed on the outcome for which to assess RoB (the first primary outcome mentioned in the results section). We did not contact study authors for additional information but used information from references provided in included articles. We assessed the RoB in duplicate and resolved disagreements via discussion. To ensure similar results between assessors, we performed one calibration exercise prior to the RoB assessment and discussed how to apply and interpret the RoB V.2.0 guidance document.^20^ We also developed additional criteria for one of the three signalling questions (online supplemental table c) and used a third author to double-check RoB judgements from baseline characteristics.

We calculated the interrater reliability for the RoB judgement using Fleiss kappa. Agreement was categorised as poor (≤0.00), slight (0.01–0.20), fair (0.21–0.40), moderate (0.41–0.60), substantial (0.61–0.80) and almost perfect (0.81–1.00) using the interpretation of the k value proposed by Landis and Koch.^21^

We used the t-test and Satterthwaite approximation for standard errors to evaluate if the mean reporting quality of articles (1) improved over time (before 2015 vs after 2015), (2) is higher in journals referring to the CONSORT statement compared with articles published in journals without a reference to it, (3) is higher for articles reporting on low RoB studies compared with (A) high RoB studies or (B) studies of some concern and (4) is lower for articles reporting on studies with an unclear RoB judgement (some concern) compared with a clear RoB judgement (high or low).

## Results

**Figure 1:**
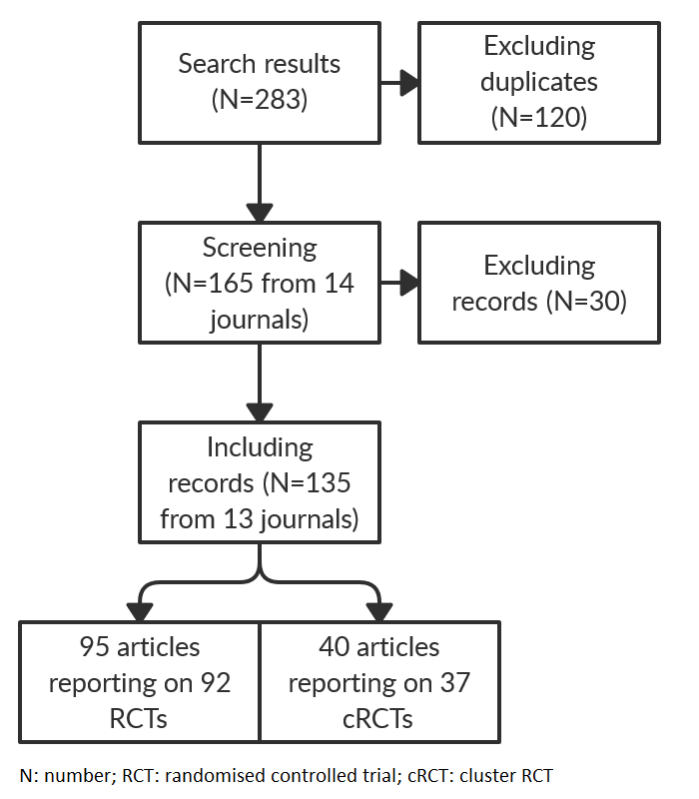
Flow chart.

Our search resulted in 135 included articles from 13 journals reporting on 129 (92 RCTs and 37 cRCTs) (figure 1, online supplemental table d). Six studies were reported in more than one article. Four studies did not provide enough information to identify the randomisation type. We categorised these as RCTs. A minority of journals (N=4) required authors to comply with the CONSORT statement (table 1). In our sample the journal with the highest mean reporting quality score was Occupational Environmental Medicine, followed by Annals of Work Exposure and Health, and Scandinavian Journal of Work Environment and Health.

**Table 1:** Characteristics of included articles and journals ranked according to mean reporting quality

Journal	Total # articles (RCTs/cRCTs)	Impact factor 2017*	CONSORT requirement†	Mean reporting quality (%) (95 % CI)
Medycyna Pracy	1 (1/0)	0.6	No	0 (–)
International Journal of Occupational and Environmental Health‡	1 (0/1)	1.2	No‡	0 (–)
Occupational Medicine	2 (1/1)	1.5	No	0 (–)
Toxicology & Industrial Health	1 (1/0)	1.3	Yes	0 (–)
International Journal of Occupational Medicine and Environmental Health	2 (2/0)	1.4	No	12.5 (−12.2 to 37.2)
American Journal of Industrial Medicine	4 (4/0)	1.7	No	12.5 (−12.2 to 37.2)
Journal of Occupational and Environmental Medicine	37 (29/8)	1.4	No	17.6 (11.0 to 24.2)
Journal of Occupational Health	11 (8/3)	1.3	No	31.8 (16.4 to 47.2)
International Archives of Occupational and Environmental Health	15 (9/6)	2.1	Yes	38.3 (24.4 to 52.3)
Industrial Health	5 (3/2)	1.1	No	42.5 (13.7 to 71.3)
Scandinavian Journal of Work. Environment & Health	31 (20/11)	2.8	Yes	50.4 (39.7 to 61.1)
Annals of work Exposure and Health§	1 (0/1)	1.6	No	62.5 (–)
Occupational and Environmental Medicine	24 (17/7)	4	Yes	65.1 (51.2 to 79.0)
All journals (N=13)	135 (95/40)	–	–	37.4 (31.9 to 43.0)

*As of June 2020

†Instructions to authors require authors to comply with CONSORT statement or checklist.

‡Gone out of print.

§Previously Annals of Occupational Hygiene.

CONSORT, Consolidated Standards of Reporting Trials; cRCTs, cluster randomised controlled trials; RCTs, randomised controlled trials.

Reporting quality was slightly better for RCTs (mean 38.9, SD 34.2) than cRCTs (mean 33.8, SD 28.8). All required checklist items were reported in 10% of the articles (13/135). Most articles (78/135) reported less than half of the checklist items (online supplemental table e). Sequence generation methods were most frequently reported (checklist item 8a) and the implementation of randomisation the least (checklist item 10 and 10a, b, c) (table 2, online supplemental table e). In some articles authors reported both the method and type of sequence generation (34 RCTs, 7 cRCTs) in half of those articles either allocation concealment method (18 RCTs, 4 cRCTs) or the implementation of the randomisation (14 RCTs, 2 cRCTs) were also reported.

**Table 2:** Checklist items reported by articles of RCTs and cRCTs

CONSORT checklist items reported in N (%) articles	RCTs (N=95)	cRCTs (N=40)	Total (N=135)
All items	12 (13%)	1 (3%)	13 (10%)
Sequence generation method (8a)	54 (59%)	16 (42%)	70 (54%)
Sequence generation type	46 (53%)	9 (28%)	55 (41%)
Type of randomisation (8b)	46 (53%)	13 (34%)	59 (47%)
Details of stratification or matching (extension 8b)	na	15 (45%)	15 (14%)
Allocation concealment	27 (32%)	8 (20%)	35 (26%)
Mechanisms to conceal sequence until allocation (9)	27 (32%)	10 (26%)	37 (30%)
Concealment at cluster or individual level (extension 9)	na	12 (34%)	12 (11%)
Implementation of randomisation	21 (26%)	2 (3%)	23 (17%)
Who generated sequence, who enrolled, who assigned (10)	21 (26%)	na	21 (18%)
Who generated sequence, who enrolled clusters, who assigned (10a)	na	6 (15%)	6 (5%)
How were individual participants included in clusters (10b)	na	17 (43%)	17 (13%)
Who gave consent and when (10c)	na	19 (44%)	19 (14%)

CONSORT, Consolidated Standards of Reporting Trials; cRCTs, cluster randomised controlled trial; na, not applicable; RCTs, randomised controlled trial.

The risk of selection bias was low in 49 trials (52 articles), of some concern for almost half the trials (63 studies reported in 66 articles), and high in 17 trials (17 articles).

The value for fixed-marginal kappa showed a moderate strength of agreement in RoB assessments between assessors (0.50; 95% CI 0.36 to 0.64).

### Reporting quality and CONSORT requirement in journals

Reporting quality in articles published in journals requiring CONSORT compliance was on average 31% points higher than articles published in journals without a requirement (table 3).

**Table 3:** Differences in the average reporting quality in articles between journals, year of publication and RoB in studies

Average reporting quality in articles	# articles	Mean reporting quality (%) (95% CI)	Mean difference (95% CI)	P value
Journals with versus without CONSORT requirement				
With CONSORT requirement	71	52.1 (44.4 to 59.8)		
Without CONSORT requirement	64	21.1 (15.1 to 27.1)	31.0 (21.4 to 40.7)	<0.001
Older versus newer publications				
Articles published 2010–2014	69	34.6 (26.7 to 42.6)		
Articles published 2015–2019	66	40.3 (32.4 to 48.2)	−5.7 (−16.8 to 5.4)	0.309
Articles of low versus some concern vs high risk of bias studies				
Low	52	61.1 (53.2 to 68.9)		
Some concern	66	21.2 (15.2 to 27.3)	39.8 (30.0 to 49.7)	<0.001
High	17	27.9 (12.4 to 43.4)	33.1 (16.1 to 50.2)	<0.001
Articles of unclear versus clear risk of bias studies				
Unclear RoB (some concern)	66	21.2 (15.2 to 27.3)		
Clear RoB (low or high)	69	52.9 (45.2 to 60.6)	−31.7 (−41.4 to −22.0)	<0.001

CONSORT, Consolidated Standards of Reporting Trials; RoB, risk of bias.

### Reporting quality and selection bias

We found a clear difference in the reporting quality of trials of different risk of selection bias (table 3). The average CONSORT reporting quality for low RoB trials was 33% points higher than for high RoB trials, and 40% points higher than for trials with a RoB of some concern (p<0.001). On average trials with a clear RoB (low or high) had a 32% points higher reporting quality than trials with an unclear RoB (some concern) (p<0.001).

### Time trend in reporting quality

Articles published before and after 2015 were on average of similar reporting quality (table 3).

## Discussion

The average reporting quality in OHS trials was measured as 37.4% (95% CI 31.9% to 43.0%) compliance with the CONSORT checklist items 8–10. Articles published in journals requiring CONSORT compliance reported on average on 31% more checklist items, than those published in journals without a CONSORT requirement (p<0.001). Low RoB studies showed on average the highest reporting quality in articles (61%), which was about twice as much compared with high RoB studies (28%) and three times higher to studies with some concern (21%). Articles of studies with unclear RoB judgements (some concern) reported on average 32% less CONSORT checklist items than articles of studies with a clear RoB (high or low) (p<0.001). We did not find a trend over time in reporting quality.

Our results show an average reporting quality far below 100% compliance with CONSORT for low, some concern and high RoB studies. Results do not reflect how much compliance with CONSORT is necessary to judge the RoB in studies. Reporting quality refers to the CONSORT compliance per article, whereas the RoB is judged per study. In some cases, this meant we combined information from multiple publications to judge the RoB in one trial. Authors of Cochrane systematic reviews are advised to access trial protocols and other unpublished data, ‘to clarify incompletely reported information or understand discrepant information available in different sources’,^22^ which means considerable effort for reviewers.^23^

We found that more information is needed to judge a low compared with high RoB in trials. A trial can be of high RoB based on one item (allocation was not concealed), whereas for a low RoB judgement, information on both the allocation concealment and random sequence generation are necessary. Missing information requires judgements from baseline differences, which is more complicated. Complete compliance with CONSORT is therewith important to distinguish between low and high RoB in studies, avoid unclear RoB judgements, and save resources in the systematic review process.

The revised CONSORT statement was published in 2010. While we included trials published over a considerably long timespan (2010–2019), we did not find a difference in reporting quality between trials published during 2010–2014 and later years (2015–2019). This shows that compliance with the minimum standard set of items to be reported in trials is very unlikely to improve over time without further action.

Randomisation and allocation concealment in trials is not easy to perform and to describe. Errors can lead to biased estimate of the treatment effect or lower the quality of the evidence.^5–7^ Central randomisation and allocation procedures are less likely to be compromised compared with local randomisation, for example, with envelopes.^24^ For small trials, it is possible to use simple, free of charge on-line tools (eg, app.studyrandomizer.com or sealedenvelope.com). They provide more than a list of random numbers only and might be easier to describe.

### Strength and limitations

To our knowledge, this is the first study to review the reporting quality in OHS trials and to assess the link to three possibly related factors: CONSORT requirements of OHS journals, risk of selection bias and year of publication.

Our search is systematic and reproducible although not comprehensive. Searching one database only might have limited the number of search results and excluded trial reports published in OHS journals not indexed in MEDLINE. However, we searched for trials published in 18 different OHS journals and had a sufficiently large sample size to detect at least a 16% mean difference in CONSORT compliance rates between two groups. We believe our sample from 18 representative OHS journals make our findings reliable and applicable to the OHS field.

A limitation in our work is that we restricted reporting quality and RoB assessment to the randomisation and allocation process. This is because we consider selection bias to be a key issue in OHS, where trials are set in workplaces and are not straight forward to conduct. This was also found as a key issue in other fields.^25^

During piloting we found that interpretation of the CONSORT checklist and RoB V.2.0 tool varied between assessors and needed considerable discussion before consensus was reached. We made considerable efforts to ensure reliable judgements that can be reproduced by assessing studies in at least duplicate and by developing and applying prespecified criteria to judge the reporting quality and RoB in studies. However, it is possible that another group of researchers could reach different judgements for some trials included in our sample. The CONSORT website (www.consort-statement.org) provides many examples of good and bad trial reporting. We found this a helpful tool to better understand the checklist items, make judgements and reach consensus when necessary. Until now, exemplary guidance on the Cochrane RoB V.2.0 tool is very limited. The interrater reliability of the tool has been previously studied with lower agreement ratings (fair) compared with ours (moderate).^26^ Authors described similar difficulties for judging RoB from baseline imbalances but did not perform a calibration exercise nor develop additional criteria before applying the tool, which could explain our better rating. Our results show the need for more explicit guidance with practice examples to help reviewers applying the ROB V.2.0 tool.

We compared the reporting quality in journals with and without CONSORT requirement, based on the information provided in the submission guidelines. While the operationalisation of reporting guideline adherence may show a wide variation across journals, we believe journals will show a significant different approach in reviewing submissions compared with journals without instructions to authors to comply with CONSORT.

### Comparison with other studies

Previous studies analysed the reporting quality of trials in other medical fields than OHS.^11–13 27^ The most comprehensive was a 2012 Cochrane review,^27^ including 16 604 articles of RCTs published in general medical journals. Review authors found similar results to ours: reporting quality of trial reports was better in CONSORT endorsing journals, but the average reporting of CONSORT checklist items was found to be insufficient. Our findings reiterate that the reporting quality of trials is still suboptimal. Preferably all journals should require CONSORT compliance, including the OHS field.

While reporting guidelines are not intended to improve trial conduct, our results show that optimally randomised studies are on average better reported. Also, studies with a clear RoB assessment (high or low) were significantly better reported than studies with an unclear assessment (some concern). This shows that better reported trials enable a clear RoB judgement rather than resulting in an RoB assessment of some concern. Few studies have analysed the association between RoB and CONSORT compliance in trial reports.^25 28^ Studies in other medical fields showed similar findings to our study, in that low ROB was associated with better reporting.

## Conclusion

Reporting quality of randomisation and allocation methods in OHS trials did not improve over the last ten years. Optimally randomised trials (low RoB) and trials published in CONSORT-requiring journals are on average better reported but very few articles reach full compliance with CONSORT. Poor compliance due to omission of information hinder identification of low and high RoB in studies.

Concerted efforts are needed by journals as well as authors to consistently implement the CONSORT checklist during the planning, writing and reviewing OHS trials. Trial authors and reviewers are advised to consult the CONSORT example database to better understand checklist items. Reporting of the implementation of the random allocation sequence needs improvement: which mechanism were used to implement the sequence, who generated the sequence, who enrolled participants, and who assigned participants to interventions.^9 19^ We strongly recommend the use of a central unit for randomisation and allocation of study participants, for example, via online tools. The publication of more extensive study protocols by journals could also support CONSORT compliance already in an earlier stage. Editors and peer-reviewers of OHS journals are advised to require CONSORT compliance and pay attention to adequate reporting of the randomisation and allocation process in trials.

Future studies should focus on how to best disseminate the CONSORT checklist to funders, journals and trial authors. Future research should analyse the inter-rater reliability of the CONSORT checklist and ROB V.2.0 tool to help improve the usage and clarity to users. Online tools to centrally randomise and allocate study participants should be evaluated regarding accessibility and usability to support authors in choosing the best tool to perform and describe randomisation and allocation methods in trials. Future studies should analyse how journals can best operationalise reporting guideline adherence to help editors implement processes that ensure high reporting quality of OHS trials.

## Data Availability

All data relevant to the study are included in the article or uploaded as supplementary information.
