# Supplementary material for: Quality of reporting and risk of bias: a review of randomised trials in occupational health
Source: Occup Environ Med. 2021 Jun 23;78(9):691–6. doi: 10.1136/oemed-2020-107038 (PMC8380877; doi:10.1136/oemed-2020-107038)
Supplement: Supplementary data [file oemed-2020-107038supp001.pdf]

Supplemental tables (a-e)

Supplemental Table a Search strategy (May 2019)

PubMed	28.5.2019	
Query	Search	results
#1	"AMERICAN JOURNAL OF INDUSTRIAL MEDICINE"[Journal]) OR "ANNALS OF WORK EXPOSURES AND HEALTH"[Journal] OR "ARCHIVES OF ENVIRONMENTAL & OCCUPATIONAL HEALTH"[Journal]) OR "Arh Hig Rada Toksikol"[jour]) OR "INDUSTRIAL HEALTH"[Journal]) OR "INTERNATIONAL ARCHIVES OF OCCUPATIONAL AND ENVIRONMENTAL HEALTH"[Journal] OR "INTERNATIONAL JOURNAL OF OCCUPATIONAL AND ENVIRONMENTAL HEALTH"[Journal] OR "INTERNATIONAL JOURNAL OF OCCUPATIONAL MEDICINE AND ENVIRONMENTAL HEALTH"[Journal] OR "JOURNAL OF OCCUPATIONAL AND ENVIRONMENTAL HYGIENE"[Journal] OR "JOURNAL OF OCCUPATIONAL AND ENVIRONMENTAL MEDICINE"[Journal] OR "JOURNAL OF OCCUPATIONAL HEALTH"[Journal] OR "Journal of occupational medicine and toxicology (London, England)"[Journal] OR "Medycyna pracy"[Journal] OR "Occupational and environmental medicine"[Journal] OR "Occupational medicine (Oxford, England)"[Journal] OR "Safety and health at work"[Journal] OR "Scandinavian journal of work, environment & health"[Journal] OR "Toxicology and industrial health"[Journal] OR Ann Occup Hyg[Journal]	44150
#2	"randomized controlled trial"[Publication Type]	483168
#3	("2010"[Date - Publication] : "3000"[Date - Publication])	9817277
#4	#1 AND #2 AND #3	283

Supplemental Table b Summary of Consort checklist items regarding randomisation and allocation process

**Items**	**Short explanation**	**Applicable to**
sequence generation method (item 8a)	Method used to generate the random allocation sequence	RCTs, cRCTs
sequence generation type (item 8b)	Type of randomisation; details of any restriction (such as blocking and block size)	RCTs, cRCTs
Extension 8b	Details of stratification or matching if used	Only for cRCTS
allocation concealment mechanism (item 9)	Mechanism used to implement the random allocation sequence (such as sequentially numbered containers), describing any steps taken to conceal the sequence until interventions were assigned	RCTs, cRCTs
Extension 9	Specification that allocation was based on clusters rather than individuals and whether allocation concealment (if any) was at the cluster level, the individual participant level or both	Only for cRCTs
randomisation implementation (item 10)	Who generated the random allocation sequence, who enrolled participants, and who assigned participants to interventions	Only for RCTs
10a	Who generated the random allocation sequence, who enrolled clusters, and who assigned clusters to interventions	Only for cRCTS
10b	Mechanism by which individual participants were included in clusters for the purposes of the trial (such as complete enumeration, random sampling)	Only for cRCTs
10c	From whom consent was sought (representatives of the cluster, or individual cluster members, or both) and whether consent was sought before or after randomisation	Only for cRCTs

Supplemental Table c Summary of ROB tool 2.0 guidance document and our additional criteria to judge RoB from baseline differences

Do baseline differences suggest a problem with the randomization process?	instructions from the Guidance document[1]	Our extension/ interpretation	Examples
**Answer 'No' if**	no imbalances are apparent or if any observed imbalances are compatible with chance. (A small number of differences identified as 'statistically significant' at the conventional 0.05 threshold should usually be considered to be compatible with chance.)	enough information available to judge that (im)balance of baseline characteristics fits randomization methods	[22]:<br>**randomization**: cRCT, clusters by manager participants report to, clusters were paired by age and three other factors of participants<br>**outcome**: health outcome<br>**# of participants:** same number of randomized study participants and participants with baseline measures (N=116), control group = 57 and intervention group = 59<br>**statistically significant baseline differences**: reported for participants not clusters, no significant differences except for one out of 22 variables (>12yrs of education) which was not a variable for pairing,<br>**imbalance in key prognostic factors**: 1-year age difference between groups, outcome measure similar between groups<br>**judgment**: observed imbalances and similarity in baseline characteristics

			compatible with type of randomization, no information for judgement missing
**Answer 'Yes'** if any of the following	substantial differences between intervention group sizes, compared with the intended allocation ratio:<br><br>One example is a 1948 trial: Anticoagulants were administered to patients admitted on odd admission dates (n = 589) and conventional therapy to patients admitted on even admission dates (n = 442). Such a large difference in numbers is very unlikely given the expected 1:1 allocation ratio(P =0.001), raising suspicion that investigators manipulated the allocation so that more patients were admitted on odd dates so that they would receive the new anti-coagulant	no extension	If group size differences can be explained with clusters this cannot be answered with Yes, e.g. as seen in [23] group size differences (I=266 C = 255) due to clusters randomized (I=2, C=2)
	a substantial excess in statistically significant differences in baseline characteristics between intervention groups, beyond that expected by chance	see below	
	imbalance in one or more key prognostic factors, or baseline measures of outcome variables, that is very unlikely to be due to chance and for which the between-group difference is big enough	- for health outcomes: if baseline outcome and/or age differ statistically significant AND the difference is clinically relevant	

	to result in bias in the intervention effect estimate	- for other outcomes: if the baseline outcome measurements and/or another prognostic factor differ statistically significant AND the difference is clinically relevant<br><br>Note: this does not apply for trials using simple randomization with less than 200 participants (because any difference can be expected with simple randomization)	
	excessive similarity in baseline characteristics that is not compatible with chance	groups are deemed too similar if:<br><br>groups show no differences in important prognostic factors (e.g. outcome measure and age) without using mechanisms for achieving balance (e.g. minimization or stratification)	fictional example of very similar baseline in two groups would be: 49.64 (13.65) and 49.63 (12.23)<br><br>if it's not down to the decimals we would not deem it too similar, e.g. as seen in [24] age [mean (SD)] 49.64 (13.65) and 50.21 (12.23)
**Answer 'No information'** when	there is no useful baseline information available (e.g. abstracts, or studies that reported only baseline characteristics of participants in the final analysis)	baseline measurement of important prognostic factor e.g. outcome measure missing,<br><br>OR<br><br>no statistics have been provided	[24] baseline characteristics not reported for all participants that were randomized, also: statistical significance not tested, type of randomization not reported<br><br>[23] cRCT: type of randomization and

			statistical tests not reported<br><br>**imbalance in key prognostic factors**: 4 years age difference, relevant differences between groups in outcome measure (1:2 ratio of monthly working hours lost due to sick leave), information missing to judge if imbalance is due to randomization<br><br>[25]: **imbalance in key prognostic factors**: no statistically significant or clinical relevant differences for age and three other factors, but outcome measure at baseline not reported

[1] RoB2 Development Group. Revised Cochrane risk-of-bias tool for randomized trials (RoB 2). In: Higgins JP, Savović J, Page MJ, Sterne JAC (eds.) 2019. https://drive.google.com/file/d/19R9savfPdCHC8XLz2iiMvL_71lPJERWK/view (07. September 2020)

Supplemental Table d List of included articles (N=135)

Article ID	Citation
1	Ahola K, Vuori J, Toppinen-Tanner S, Mutanen P, Honkonen T. Resource-enhancing group intervention against depression at workplace: who benefits? A randomised controlled study with a 7-month follow-up. Occupational and environmental medicine. 2012;69(12):870-6.
2	Aikens KA, Astin J, Pelletier KR, Levanovich K, Baase CM, Park YY, et al. Mindfulness goes to work: impact of an online workplace intervention. Journal of occupational and environmental medicine. 2014;56(7):721-31.
3	Allexandre D, Bernstein AM, Walker E, Hunter J, Roizen MF, Morledge TJ. A Web-Based Mindfulness Stress Management Program in a Corporate Call Center: A Randomized Clinical Trial to Evaluate the Added Benefit of Onsite Group Support. Journal of occupational and environmental medicine. 2016;58(3):254-64.
4	Arbogast JW, Moore-Schiltz L, Jarvis WR, Harpster-Hagen A, Hughes J, Parker A. Impact of a Comprehensive Workplace Hand Hygiene Program on Employer Health Care Insurance Claims and Costs, Absenteeism, and Employee Perceptions and Practices. Journal of occupational and environmental medicine. 2016;58(6):e231-40.
5	Arends I, van der Klink JJ, van Rhenen W, de Boer MR, Bultmann U. Prevention of recurrent sickness absence in workers with common mental disorders: results of a cluster-randomised controlled trial. Occupational and environmental medicine. 2014;71(1):21-9.
6	Arnetz JE, Hamblin L, Russell J, Upfal MJ, Luborsky M, Janisse J, et al. Preventing Patient-to-Worker Violence in Hospitals: Outcome of a Randomized Controlled Intervention. Journal of occupational and environmental medicine. 2017;59(1):18-27.
7	Baatjies R, Meijster T, Heederik D, Sander I, Jeebhay MF. Effectiveness of interventions to reduce flour dust exposures in supermarket bakeries in South Africa. Occupational and environmental medicine. 2014;71(12):811-8.
8	Baydur H, Ergor A, Demiral Y, Akalin E. Effects of participatory ergonomic intervention on the development of upper extremity musculoskeletal disorders and disability in office employees using a computer. Journal of occupational health. 2016;58(3):297-309.
9	Becker A, Angerer P, Muller A. The prevention of musculoskeletal complaints: a randomized controlled trial on additional effects of a work-related psychosocial coaching intervention compared to physiotherapy alone. International archives of occupational and environmental health. 2017;90(4):357-71.
10	Bennett JB, Broome KM, Schwab-Pilley A, Gilmore P. A web-based approach to address cardiovascular risks in managers: results of a randomized trial. Journal of occupational and environmental medicine. 2011;53(8):911-8.
11	Beresford SA, Bishop SK, Brunner NL, Duncan GE, McGregor BA, McLerran DF, et al. Environmental assessment at worksites after a multilevel intervention to promote activity and changes in eating: the PACE project. Journal of occupational and environmental medicine. 2010;52 Suppl 1:S22-8.
12	Bian Y, Xiong H, Zhang L, Tang T, Liu Z, Xu R, et al. Change in coping strategies following intensive intervention for special-service military personnel as civil emergency responders. Journal of occupational health. 2011;53(1):36-44.
14	Bostock S, Luik AI, Espie CA. Sleep and Productivity Benefits of Digital Cognitive Behavioral Therapy for Insomnia: A Randomized Controlled Trial Conducted in the Workplace Environment. Journal of occupational and environmental medicine. 2016;58(7):683-9.
15	Bovo R, Trevisi P, Emanuelli E, Martini A. Voice amplification for primary school teachers with voice disorders: a randomized clinical trial. International journal of occupational medicine and environmental health. 2013;26(3):363-72.

16	Brown DK, Barton JL, Pretty J, Gladwell VF. Walks4Work: assessing the role of the natural environment in a workplace physical activity intervention. Scandinavian journal of work, environment & health. 2014;40(4):390-9.
17	Carvalho Mesquita C, Ribeiro JC, Moreira P. Effect of a specific exercise program on the strength and resistance levels of lumbar muscles in warehouse workers. International journal of occupational medicine and environmental health. 2012;25(1):80-8.
18	Chaleat-Valayer E, Denis A, Abelin-Genevois K, Zelmar A, Siani-Trebern F, Touzet S, et al. Long-term effectiveness of an educational and physical intervention for preventing low-back pain recurrence: a randomized controlled trial. Scandinavian journal of work, environment & health. 2016;42(6):510-9.
19	Chopp-Hurley JN, Brenneman EC, Wiebenga EG, Bulbrook B, Keir PJ, Maly MR. Randomized Controlled Trial Investigating the Role of Exercise in the Workplace to Improve Work Ability, Performance, and Patient-Reported Symptoms Among Older Workers With Osteoarthritis. Journal of occupational and environmental medicine. 2017;59(6):550-6.
20	Christensen JR, Overgaard K, Hansen K, Sogaard K, Holtermann A. Effects on presenteeism and absenteeism from a 1-year workplace randomized controlled trial among health care workers. Journal of occupational and environmental medicine. 2013;55(10):1186-90.
21	Coffeng JK, Hendriksen IJ, Duijts SF, Twisk JW, van Mechelen W, Boot CR. Effectiveness of a combined social and physical environmental intervention on presenteeism, absenteeism, work performance, and work engagement in office employees. Journal of occupational and environmental medicine. 2014;56(3):258-65.
22	Comper MLC, Dennerlein JT, Evangelista GDS, Rodrigues da Silva P, Padula RS. Effectiveness of job rotation for preventing work-related musculoskeletal diseases: a cluster randomised controlled trial. Occupational and environmental medicine. 2017;74(8):545-52.
26	de Vries JD, van Hooff ML, Guerts SA, Kompier MA. Exercise to reduce work-related fatigue among employees: a randomized controlled trial. Scandinavian journal of work, environment & health. 2017;43(4):337-49.
27	Deitz D, Cook RF, Hersch RK, Leaf S. Heart healthy online: an innovative approach to risk reduction in the workplace. Journal of occupational and environmental medicine. 2014;56(5):547-53.
28	Dellve L, Ahlstrom L, Jonsson A, Sandsjo L, Forsman M, Lindegard A, et al. Myofeedback training and intensive muscular strength training to decrease pain and improve work ability among female workers on long-term sick leave with neck pain: a randomized controlled trial. International archives of occupational and environmental health. 2011;84(3):335-46.
29	Doda D, Rothmore P, Pisaniello D, Briggs N, Stewart S, Mahmood M, et al. Relative benefit of a stage of change approach for the prevention of musculoskeletal pain and discomfort: a cluster randomised trial. Occupational and environmental medicine. 2015;72(11):784-91.
31	Driessen MT, Proper KI, Anema JR, Knol DL, Bongers PM, van der Beek AJ. The effectiveness of participatory ergonomics to prevent low-back and neck pain--results of a cluster randomized controlled trial. Scandinavian journal of work, environment & health. 2011;37(5):383-93.
32	Dropkin J, Kim H, Punnett L, Wegman DH, Warren N, Buchholz B. Effect of an office ergonomic randomised controlled trial among workers with neck and upper extremity pain. Occupational and environmental medicine. 2015;72(1):6-14.
35	Ebert DD, Heber E, Berking M, Riper H, Cuijpers P, Funk B, et al. Self-guided internet-based and mobile-based stress management for employees: results of a randomised controlled trial. Occupational and environmental medicine. 2016;73(5):315-23.

36	Ebert DD, Lehr D, Boss L, Riper H, Cuijpers P, Andersson G, et al. Efficacy of an internet-based problem-solving training for teachers: results of a randomized controlled trial. Scandinavian journal of work, environment & health. 2014;40(6):582-96.
37	Ebert DD, Lehr D, Heber E, Riper H, Cuijpers P, Berking M. Internet- and mobile-based stress management for employees with adherence-focused guidance: efficacy and mechanism of change. Scandinavian journal of work, environment & health. 2016;42(5):382-94.
38	Eguchi M, Ohta M, Yamato H. The effects of single long and accumulated short bouts of exercise on cardiovascular risks in male Japanese workers: a randomized controlled study. Industrial health. 2013;51(6):563-71.
39	Erman MK, Seiden DJ, Yang R, Dammerman R. Efficacy and tolerability of armodafinil: effect on clinical condition late in the shift and overall functioning of patients with excessive sleepiness associated with shift work disorder. Journal of occupational and environmental medicine. 2011;53(12):1460-5.
40	Esmaeilzadeh S, Ozcan E, Capan N. Effects of ergonomic intervention on work-related upper extremity musculoskeletal disorders among computer workers: a randomized controlled trial. International archives of occupational and environmental health. 2014;87(1):73-83.
41	Faghri PD, Simon J, Huedo-Medina T, Gorin A. Perceived Self-Efficacy and Financial Incentives: Factors Affecting Health Behaviors and Weight Loss in a Workplace Weight Loss Intervention. Journal of occupational and environmental medicine. 2017;59(5):453-60.
42	Fisker MH, Ebbehoj NE, Vejlstrup SG, Lindschou J, Gluud C, Winkel P, et al. Prevention of hand eczema: effect of an educational program versus treatment as usual - results of the randomized clinical PREVEX trial. Scandinavian journal of work, environment & health. 2018;44(2):212-8.
44	Framke E, Sorensen OH, Pedersen J, Rugulies R. Effect of a participatory organizational-level occupational health intervention on short-term sickness absence: a cluster randomized controlled trial. Scandinavian journal of work, environment & health. 2016;42(3):192-200.
45	Gartner FR, Nieuwenhuijsen K, Ketelaar SM, van Dijk FJ, Sluiter JK. The mental vitality @ work study: effectiveness of a mental module for workers' health surveillance for nurses and allied health care professionals on their help-seeking behavior. Journal of occupational and environmental medicine. 2013;55(10):1219-29.
46	Genin PM, Degoutte F, Finaud J, Pereira B, Thivel D, Duclos M. Effect of a 5-Month Worksite Physical Activity Program on Tertiary Employees Overall Health and Fitness. Journal of occupational and environmental medicine. 2017;59(2):e3-e10.
47	Glass N, Hanson GC, Anger WK, Laharnar N, Campbell JC, Weinstein M, et al. Computer-based training (CBT) intervention reduces workplace violence and harassment for homecare workers. American journal of industrial medicine. 2017;60(7):635-43.
48	Glass N, Hanson GC, Laharnar N, Anger WK, Perrin N. Interactive training improves workplace climate, knowledge, and support towards domestic violence. American journal of industrial medicine. 2016;59(7):538-48.
49	Glasscock DJ, Carstensen O, Dalgaard VL. Recovery from work-related stress: a randomized controlled trial of a stress management intervention in a clinical sample. International archives of occupational and environmental health. 2018;91(6):675-87.
50	Gram B, Holtermann A, Bultmann U, Sjogaard G, Sogaard K. Does an exercise intervention improving aerobic capacity among construction workers also improve musculoskeletal pain, work ability, productivity, perceived physical exertion, and sick leave?: a randomized controlled trial. Journal of occupational and environmental medicine. 2012;54(12):1520-6.

53	Gupta N, Wahlin-Jacobsen CD, Abildgaard JS, Henriksen LN, Nielsen K, Holtermann A. Effectiveness of a participatory physical and psychosocial intervention to balance the demands and resources of industrial workers: A cluster-randomized controlled trial. Scandinavian journal of work, environment & health. 2018;44(1):58-68.
54	Hagiwara Y, Yabe Y, Yamada H, Watanabe T, Kanazawa K, Koide M, et al. Effects of a wearable type lumbosacral support for low back pain among hospital workers: A randomized controlled trial. Journal of occupational health. 2017;59(2):201-9.
55	Harber P, Boumis RJ, Su J, Barrett S, Alongi G. Comparison of three respirator user training methods. Journal of occupational and environmental medicine. 2013;55(12):1484-8.
56	Hartfiel N, Burton C, Rycroft-Malone J, Clarke G, Havenhand J, Khalsa SB, et al. Yoga for reducing perceived stress and back pain at work. Occupational medicine (Oxford, England). 2012;62(8):606-12.
57	Hartfiel N, Havenhand J, Khalsa SB, Clarke G, Krayer A. The effectiveness of yoga for the improvement of well-being and resilience to stress in the workplace. Scandinavian journal of work, environment & health. 2011;37(1):70-6.
59	Hees HL, de Vries G, Koeter MW, Schene AH. Adjuvant occupational therapy improves long-term depression recovery and return-to-work in good health in sick-listed employees with major depression: results of a randomised controlled trial. Occupational and environmental medicine. 2013;70(4):252-60.
60	Hellstrom L, Bech P, Hjorthoj C, Nordentoft M, Lindschou J, Eplov LF. Effect on return to work or education of Individual Placement and Support modified for people with mood and anxiety disorders: results of a randomised clinical trial. Occupational and environmental medicine. 2017;74(10):717-25.
61	Hogg-Johnson S, Robson L, Cole DC, Amick BC, 3rd, Tompa E, Smith PM, et al. A randomised controlled study to evaluate the effectiveness of targeted occupational health and safety consultation or inspection in Ontario manufacturing workplaces. Occupational and environmental medicine. 2012;69(12):890-900.
62	Hong O, Chin DL, Fiola LA, Kazanis AS. The effect of a booster intervention to promote hearing protection behavior in operating engineers. American journal of industrial medicine. 2013;56(2):258-66.
64	Hutting N, Staal JB, Engels JA, Heerkens YF, Detaille SI, Nijhuis-van der Sanden MW. Effect evaluation of a self-management programme for employees with complaints of the arm, neck or shoulder: a randomised controlled trial. Occupational and environmental medicine. 2015;72(12):852-61.
66	Jakobsen MD, Sundstrup E, Brandt M, Jay K, Aagaard P, Andersen LL. Effect of workplace-versus home-based physical exercise on musculoskeletal pain among healthcare workers: a cluster randomized controlled trial. Scandinavian journal of work, environment & health. 2015;41(2):153-63.
67	Jay K, Frisch D, Hansen K, Zebis MK, Andersen CH, Mortensen OS, et al. Kettlebell training for musculoskeletal and cardiovascular health: a randomized controlled trial. Scandinavian journal of work, environment & health. 2011;37(3):196-203.
68	Jensen LD, Maribo T, Schiottz-Christensen B, Madsen FH, Gonge B, Christensen M, et al. Counselling low-back-pain patients in secondary healthcare: a randomised trial addressing experienced workplace barriers and physical activity. Occupational and environmental medicine. 2012;69(1):21-8.
69	Justesen JB, Sogaard K, Dalager T, Christensen JR, Sjogaard G. The Effect of Intelligent Physical Exercise Training on Sickness Presenteeism and Absenteeism Among Office Workers. Journal of occupational and environmental medicine. 2017;59(10):942-8.
70	Kajiki S, Izumi H, Hayashida K, Kusumoto A, Nagata T, Mori K. A randomized controlled trial of the effect of participatory ergonomic low back pain training on workplace improvement. Journal of occupational health. 2017;59(3):256-66.

71	Kaku A, Nishinoue N, Takano T, Eto R, Kato N, Ono Y, et al. Randomized controlled trial on the effects of a combined sleep hygiene education and behavioral approach program on sleep quality in workers with insomnia. Industrial health. 2012;50(1):52-9.
72	Kasperczyk S, Dobrakowski M, Kasperczyk A, Romuk E, Rykaczewska-Czerwinska M, Pawlas N, et al. Effect of N-acetylcysteine administration on homocysteine level, oxidative damage to proteins, and levels of iron (Fe) and Fe-related proteins in lead-exposed workers. Toxicology and industrial health. 2016;32(9):1607-18.
73	Kasperczyk S, Dobrakowski M, Kasperczyk J, Romuk E, Prokopowicz A, Birkner E. The influence of beta-carotene on homocysteine level and oxidative stress in lead-exposed workers. Medycyna pracy. 2014;65(3):309-16.
74	Kawashima M, Sano K, Takechi S, Tsubota K. Impact of lifestyle intervention on dry eye disease in office workers: a randomized controlled trial. Journal of occupational health. 2018;60(4):281-8.
75	Ketelaar SM, Nieuwenhuijsen K, Gartner FR, Bolier L, Smeets O, Sluiter JK. Mental Vitality @ Work: The effectiveness of a mental module for workers' health surveillance for nurses and allied health professionals, comparing two approaches in a cluster-randomised controlled trial. International archives of occupational and environmental health. 2014;87(5):527-38.
77	Kimura R, Mori M, Tajima M, Somemura H, Sasaki N, Yamamoto M, et al. Effect of a brief training program based on cognitive behavioral therapy in improving work performance: A randomized controlled trial. Journal of occupational health. 2015;57(2):169-78.
78	Korshoj M, Lidegaard M, Skotte JH, Krustrup P, Krause N, Sogaard K, et al. Does aerobic exercise improve or impair cardiorespiratory fitness and health among cleaners? A cluster randomized controlled trial. Scandinavian journal of work, environment & health. 2015;41(2):140-52.
79	Korshoj M, Ravn MH, Holtermann A, Hansen AM, Krustrup P. Aerobic exercise reduces biomarkers related to cardiovascular risk among cleaners: effects of a worksite intervention RCT. International archives of occupational and environmental health. 2016;89(2):239-49.
80	Kouwenhoven-Pasmooij TA, Robroek SJ, Nieboer D, Helmhout PH, Wery MF, Hunink M, et al. Quality of motivational interviewing matters: the effect on participation in health-promotion activities in a cluster randomized controlled trial. Scandinavian journal of work, environment & health. 2018;44(4):414-22.
81	Kramer MK, Molenaar DM, Arena VC, Venditti EM, Meehan RJ, Miller RG, et al. Improving employee health: evaluation of a worksite lifestyle change program to decrease risk factors for diabetes and cardiovascular disease. Journal of occupational and environmental medicine. 2015;57(3):284-91.
82	Lammerts L, Schaafsma FG, Bonefaas-Groenewoud K, van Mechelen W, Anema J. Effectiveness of a return-to-work program for workers without an employment contract, sick-listed due to common mental disorders. Scandinavian journal of work, environment & health. 2016;42(6):469-80.
84	Lenderink AF, Spreeuwers D, van der Klink JJ, van Dijk FJ. Information and feedback to improve occupational physicians' reporting of occupational diseases: a randomised controlled trial. International archives of occupational and environmental health. 2010;83(4):381-8.
85	Lerner D, Adler D, Hermann RC, Chang H, Ludman EJ, Greenhill A, et al. Impact of a work-focused intervention on the productivity and symptoms of employees with depression. Journal of occupational and environmental medicine. 2012;54(2):128-35.
86	Lexis MA, Jansen NW, Huibers MJ, van Amelsvoort LG, Berkouwer A, Tjin ATG, et al. Prevention of long-term sickness absence and major depression in high-risk employees: a randomised controlled trial. Occupational and environmental medicine. 2011;68(6):400-7.

87	Li X, Lin C, Liu C, Ke S, Wan Q, Luo H, et al. Comparison of the effectiveness of resistance training in women with chronic computer-related neck pain: a randomized controlled study. International archives of occupational and environmental health. 2017;90(7):673-83.
88	Lidegaard M, Sogaard K, Krustrup P, Holtermann A, Korshoj M. Effects of 12 months aerobic exercise intervention on work ability, need for recovery, productivity and rating of exertion among cleaners: a worksite RCT. International archives of occupational and environmental health. 2018;91(2):225-35.
89	Limm H, Gundel H, Heinmuller M, Marten-Mittag B, Nater UM, Siegrist J, et al. Stress management interventions in the workplace improve stress reactivity: a randomised controlled trial. Occupational and environmental medicine. 2011;68(2):126-33.
91	Mainsbridge CP, Cooley PD, Fraser SP, Pedersen SJ. The effect of an e-health intervention designed to reduce prolonged occupational sitting on mean arterial pressure. Journal of occupational and environmental medicine. 2014;56(11):1189-94.
92	Martimo KP, Shiri R, Miranda H, Ketola R, Varonen H, Viikari-Juntura E. Effectiveness of an ergonomic intervention on the productivity of workers with upper-extremity disorders--a randomized controlled trial. Scandinavian journal of work, environment & health. 2010;36(1):25-33.
93	Matsugaki R, Kuhara S, Saeki S, Jiang Y, Michishita R, Ohta M, et al. Effectiveness of workplace exercise supervised by a physical therapist among nurses conducting shift work: A randomized controlled trial. Journal of occupational health. 2017;59(4):327-35.
94	McCauley L, Runkle JD, Samples J, Williams B, Muniz JF, Semple M, et al. Oregon indigenous farmworkers: results of promotor intervention on pesticide knowledge and organophosphate metabolite levels. Journal of occupational and environmental medicine. 2013;55(10):1164-70.
95	Melton L, Anfield R, Kane G, White N, Young J, Dunnington K. Reducing the incidence of short-term disability: testing the effectiveness of an absence prediction and prevention intervention using an experimental design. Journal of occupational and environmental medicine. 2012;54(12):1441-6.
97	Michishita R, Jiang Y, Ariyoshi D, Yoshida M, Moriyama H, Yamato H. The practice of active rest by workplace units improves personal relationships, mental health, and physical activity among workers. Journal of occupational health. 2017;59(2):122-30.
98	Morgan PJ, Collins CE, Plotnikoff RC, Cook AT, Berthon B, Mitchell S, et al. The impact of a workplace-based weight loss program on work-related outcomes in overweight male shift workers. Journal of occupational and environmental medicine. 2012;54(2):122-7.
100	Muschalla B. Negative work perception not changed in a short work-anxiety-coping group therapy intervention. International journal of occupational and environmental health. 2016;22(4):321-4.
101	Nakamura S, Somemura H, Sasaki N, Yamamoto M, Tanaka M, Tanaka K. Effect of management training in organizational justice: a randomized controlled trial. Industrial health. 2016;54(3):263-71.
102	Nanri A, Tomita K, Matsushita Y, Ichikawa F, Yamamoto M, Nagafuchi Y, et al. Effect of six months lifestyle intervention in Japanese men with metabolic syndrome: randomized controlled trial. Journal of occupational health. 2012;54(3):215-22.
103	Nishinoue N, Takano T, Kaku A, Eto R, Kato N, Ono Y, et al. Effects of sleep hygiene education and behavioral therapy on sleep quality of white-collar workers: a randomized controlled trial. Industrial health. 2012;50(2):123-31.
104	Noordik E, van der Klink JJ, Geskus RB, de Boer MR, van Dijk FJ, Nieuwenhuijsen K. Effectiveness of an exposure-based return-to-work program for workers on sick leave due to common mental disorders: a cluster-randomized controlled trial. Scandinavian journal of work, environment & health. 2013;39(2):144-54.

105	Ognibene GT, Torres W, von Eyben R, Horst KC. Impact of a Sit-Stand Workstation on Chronic Low Back Pain: Results of a Randomized Trial. Journal of occupational and environmental medicine. 2016;58(3):287-93.
106	Ostbye T, Stroo M, Brouwer RJ, Peterson BL, Eisenstein EL, Fuemmeler BF, et al. Steps to Health employee weight management randomized control trial: short-term follow-up results. Journal of occupational and environmental medicine. 2015;57(2):188-95.
107	Oude Hengel KM, Blatter BM, van der Molen HF, Bongers PM, van der Beek AJ. The effectiveness of a construction worksite prevention program on work ability, health, and sick leave: results from a cluster randomized controlled trial. Scandinavian journal of work, environment & health. 2013;39(5):456-67.
108	Ozaki I, Watai I, Nishijima M, Saito N. Randomized controlled trial of Web-based weight-loss intervention with human support for male workers under 40. Journal of occupational health. 2019;61(1):110-20.
109	Patterson PD, Buysse DJ, Weaver MD, Doman JM, Moore CG, Suffoletto BP, et al. Real-time fatigue reduction in emergency care clinicians: The SleepTrackTXT randomized trial. American journal of industrial medicine. 2015;58(10):1098-113.
110	Poulsen OM, Aust B, Bjorner JB, Rugulies R, Hansen JV, Tverborgvik T, et al. Effect of the Danish return-to-work program on long-term sickness absence: results from a randomized controlled trial in three municipalities. Scandinavian journal of work, environment & health. 2014;40(1):47-56.
111	Puhkala J, Kukkonen-Harjula K, Mansikkamaki K, Aittasalo M, Hublin C, Karmeniemi P, et al. Lifestyle counseling to reduce body weight and cardiometabolic risk factors among truck and bus drivers--a randomized controlled trial. Scandinavian journal of work, environment & health. 2015;41(1):54-64.
112	Quach T, Von Behren J, Tsoh J, Reynolds P, Fu L, Nguyen T, et al. Improving the knowledge and behavior of workplace chemical exposures in Vietnamese-American nail salon workers: a randomized controlled trial. International archives of occupational and environmental health. 2018;91(8):1041-50.
113	Rantonen J, Luoto S, Vehtari A, Hupli M, Karppinen J, Malmivaara A, et al. The effectiveness of two active interventions compared to self-care advice in employees with non-acute low back symptoms: a randomised, controlled trial with a 4-year follow-up in the occupational health setting. Occupational and environmental medicine. 2012;69(1):12-20.
114	Rantonen J, Vehtari A, Karppinen J, Luoto S, Viikari-Juntura E, Hupli M, et al. Face-to-face information combined with a booklet versus a booklet alone for treatment of mild low-back pain: a randomized controlled trial. Scandinavian journal of work, environment & health. 2014;40(2):156-66.
115	Rasotto C, Bergamin M, Sieverdes JC, Gobbo S, Alberton CL, Neunhaeuserer D, et al. A tailored workplace exercise program for women at risk for neck and upper limb musculoskeletal disorders: a randomized controlled trial. Journal of occupational and environmental medicine. 2015;57(2):178-83.
116	Reme SE, Grasdal AL, Lovvik C, Lie SA, Overland S. Work-focused cognitive-behavioural therapy and individual job support to increase work participation in common mental disorders: a randomised controlled multicentre trial. Occupational and environmental medicine. 2015;72(10):745-52.
118	Salomonsson S, Santoft F, Lindsater E, Ejeby K, Ljotsson B, Ost LG, et al. Cognitive-behavioural therapy and return-to-work intervention for patients on sick leave due to common mental disorders: a randomised controlled trial. Occupational and environmental medicine. 2017;74(12):905-12.
119	Sanaeinasab H, Saffari M, Valipour F, Alipour HR, Sepandi M, Al Zaben F, et al. The effectiveness of a model-based health education intervention to improve ergonomic

	posture in office computer workers: a randomized controlled trial. International archives of occupational and environmental health. 2018;91(8):951-62.
120	Sasaki N, Somemura H, Nakamura S, Yamamoto M, Isojima M, Shinmei I, et al. Effects of Brief Communication Skills Training for Workers Based on the Principles of Cognitive Behavioral Therapy: A Randomized Controlled Trial. Journal of occupational and environmental medicine. 2017;59(1):61-6.
122	Schiller H, Soderstrom M, Lekander M, Rajaleid K, Kecklund G. A randomized controlled intervention of workplace-based group cognitive behavioral therapy for insomnia. International archives of occupational and environmental health. 2018;91(4):413-24.
123	Schuna JM, Jr., Swift DL, Hendrick CA, Duet MT, Johnson WD, Martin CK, et al. Evaluation of a workplace treadmill desk intervention: a randomized controlled trial. Journal of occupational and environmental medicine. 2014;56(12):1266-76.
124	Sforzo GA, Kaye MP, Calleri D, Ngai N. Free choice access to multipoint wellness education and related services positively impacts employee wellness: a randomized and controlled trial. Journal of occupational and environmental medicine. 2012;54(4):471-7.
127	Sihawong R, Janwantanakul P, Jiamjarasrangsi W. Effects of an exercise programme on preventing neck pain among office workers: a 12-month cluster-randomised controlled trial. Occupational and environmental medicine. 2014;71(1):63-70.
128	Sletten TL, Ftouni S, Nicholas CL, Magee M, Grunstein RR, Ferguson S, et al. Randomised controlled trial of the efficacy of a blue-enriched light intervention to improve alertness and performance in night shift workers. Occupational and environmental medicine. 2017;74(11):792-801.
129	Smits PB, de Graaf L, Radon K, de Boer AG, Bos NR, van Dijk FJ, et al. Case-based e-learning to improve the attitude of medical students towards occupational health, a randomised controlled trial. Occupational and environmental medicine. 2012;69(4):280-3.
130	Sparer EH, Catalano PJ, Herrick RF, Dennerlein JT. Improving safety climate through a communication and recognition program for construction: a mixed methods study. Scandinavian journal of work, environment & health. 2016;42(4):329-37.
131	Steinberg G, Scott A, Honcz J, Spettell C, Pradhan S. Reducing Metabolic Syndrome Risk Using a Personalized Wellness Program. Journal of occupational and environmental medicine. 2015;57(12):1269-74.
132	Strijk JE, Proper KI, van Mechelen W, van der Beek AJ. Effectiveness of a worksite lifestyle intervention on vitality, work engagement, productivity, and sick leave: results of a randomized controlled trial. Scandinavian journal of work, environment & health. 2013;39(1):66-75.
133	Sundstrup E, Jakobsen MD, Brandt M, Jay K, Persson R, Aagaard P, et al. Workplace strength training prevents deterioration of work ability among workers with chronic pain and work disability: a randomized controlled trial. Scandinavian journal of work, environment & health. 2014;40(3):244-51.
134	Svensson AL, Marott JL, Suadicani P, Mortensen OS, Ebbehoj NE. Sickness absence in student nursing assistants following a preventive intervention programme. Occupational medicine (Oxford, England). 2011;61(1):57-61.
137	Thiart H, Lehr D, Ebert DD, Berking M, Riper H. Log in and breathe out: internet-based recovery training for sleepless employees with work-related strain - results of a randomized controlled trial. Scandinavian journal of work, environment & health. 2015;41(2):164-74.
139	Toppinen-Tanner S, Bockerman P, Mutanen P, Martimo KP, Vuori J. Preventing Sickness Absence With Career Management Intervention: A Randomized Controlled Field Trial. Journal of occupational and environmental medicine. 2016;58(12):1202-6.
140	Uchiyama A, Odagiri Y, Ohya Y, Takamiya T, Inoue S, Shimomitsu T. Effect on mental health of a participatory intervention to improve psychosocial work environment: a cluster

	randomized controlled trial among nurses. Journal of occupational health. 2013;55(3):173-83.
141	Umanodan R, Shimazu A, Minami M, Kawakami N. Effects of computer-based stress management training on psychological well-being and work performance in japanese employees: a cluster randomized controlled trial. Industrial health. 2014;52(6):480-91.
142	Unterbrink T, Pfeifer R, Krippeit L, Zimmermann L, Rose U, Joos A, et al. Burnout and effort-reward imbalance improvement for teachers by a manual-based group program. International archives of occupational and environmental health. 2012;85(6):667-74.
143	Valley MA, Stallones L. Effect of Mindfulness-Based Stress Reduction Training on Health Care Worker Safety: A Randomized Waitlist Controlled Trial. Journal of occupational and environmental medicine. 2017;59(10):935-41.
145	van der Meer EW, Boot CR, Twisk JW, Coenraads PJ, Jungbauer FH, van der Gulden JW, et al. Hands4U: the effectiveness of a multifaceted implementation strategy on behaviour related to the prevention of hand eczema-a randomised controlled trial among healthcare workers. Occupational and environmental medicine. 2014;71(7):492-9.
146	van Deurssen E, Meijster T, Oude Hengel KM, Boessen R, Spaan S, Tielemans E, et al. Effectiveness of a Multidimensional Randomized Control Intervention to Reduce Quartz Exposure Among Construction Workers. The Annals of occupational hygiene. 2015;59(8):959-71.
147	van Drongelen A, Boot CR, Hlobil H, Twisk JW, Smid T, van der Beek AJ. Evaluation of an mHealth intervention aiming to improve health-related behavior and sleep and reduce fatigue among airline pilots. Scandinavian journal of work, environment & health. 2014;40(6):557-68.
148	van Oostrom SH, van Mechelen W, Terluin B, de Vet HC, Knol DL, Anema JR. A workplace intervention for sick-listed employees with distress: results of a randomised controlled trial. Occupational and environmental medicine. 2010;67(9):596-602.
149	van Wier MF, Dekkers JC, Hendriksen IJ, Heymans MW, Ariens GA, Pronk NP, et al. Effectiveness of phone and e-mail lifestyle counseling for long term weight control among overweight employees. Journal of occupational and environmental medicine. 2011;53(6):680-6.
150	Varekamp I, Verbeek JH, de Boer A, van Dijk FJ. Effect of job maintenance training program for employees with chronic disease - a randomized controlled trial on self-efficacy, job satisfaction, and fatigue. Scandinavian journal of work, environment & health. 2011;37(4):288-97.
151	Verweij LM, Proper KI, Weel AN, Hulshof CT, van Mechelen W. Long-term effects of an occupational health guideline on employees' body weight-related outcomes, cardiovascular disease risk factors, and quality of life: results from a randomized controlled trial. Scandinavian journal of work, environment & health. 2013;39(3):284-94.
152	Verweij LM, Proper KI, Weel AN, Hulshof CT, van Mechelen W. The application of an occupational health guideline reduces sedentary behaviour and increases fruit intake at work: results from an RCT. Occupational and environmental medicine. 2012;69(7):500-7.
153	Viester L, Verhagen EA, Bongers PM, van der Beek AJ. The effect of a health promotion intervention for construction workers on work-related outcomes: results from a randomized controlled trial. International archives of occupational and environmental health. 2015;88(6):789-98.
154	Viikari-Juntura E, Kausto J, Shiri R, Kaila-Kangas L, Takala EP, Karppinen J, et al. Return to work after early part-time sick leave due to musculoskeletal disorders: a randomized controlled trial. Scandinavian journal of work, environment & health. 2012;38(2):134-43.
155	Vilela BL, Benedito Silva AA, de Lira CA, Andrade Mdos S. Workplace exercise and educational program for improving fitness outcomes related to health in workers: a

	randomized controlled trial. Journal of occupational and environmental medicine. 2015;57(3):235-40.
156	Willert MV, Thulstrup AM, Bonde JP. Effects of a stress management intervention on absenteeism and return to work--results from a randomized wait-list controlled trial. Scandinavian journal of work, environment & health. 2011;37(3):186-95.
157	Wilson MG, DeJoy DM, Vandenberg RJ, Corso P, Padilla H, Zuercher H. Effect of Intensity and Program Delivery on the Translation of Diabetes Prevention Program to Worksites: A Randomized Controlled Trial of Fuel Your Life. Journal of occupational and environmental medicine. 2016;58(11):1113-20.
158	Vlasveld MC, van der Feltz-Cornelis CM, Ader HJ, Anema JR, Hoedeman R, van Mechelen W, et al. Collaborative care for sick-listed workers with major depressive disorder: a randomised controlled trial from the Netherlands Depression Initiative aimed at return to work and depressive symptoms. Occupational and environmental medicine. 2013;70(4):223-30.
159	von Thiele Schwarz U, Augustsson H, Hasson H, Stenfors-Hayes T. Promoting employee health by integrating health protection, health promotion, and continuous improvement: a longitudinal quasi-experimental intervention study. Journal of occupational and environmental medicine. 2015;57(2):217-25.
162	Yu IT, Yu W, Li Z, Qiu H, Wan S, Xie S, et al. Effectiveness of participatory training in preventing accidental occupational injuries: a randomized-controlled trial in China. Scandinavian journal of work, environment & health. 2017;43(3):226-33.
163	Yu W, Yu IT, Wang X, Li Z, Wan S, Qiu H, et al. Effectiveness of participatory training for prevention of musculoskeletal disorders: a randomized controlled trial. International archives of occupational and environmental health. 2013;86(4):431-40.

Supplemental Table e Overview reporting quality of CONSORT checklist items per article

article IDs	Sequence generation method (item 8a)	Sequence generation type (item 8b)	Extension 8b for cRCTs	Allocation concealment mechanism (item 9)	Extension 9 for cRCTs	Randomisation implementation (item 10)	Extension for cRCTs, randomisation implementation 10a: who did what	Extension for cRCTs, randomisation implementation 10b: how clusters included (all?, random?)	Extension for cRCTs, randomisation implementation 10c: Informed consent from whom and when
**Randomised controlled trials (RCT)**									
18	Yes	Yes	na	Yes	na	Yes	na	na	na
32	Yes	Yes	na	Yes	na	Yes	na	na	na
61	Yes	Yes	na	Yes	na	Yes	na	na	na
64	Yes	Yes	na	Yes	na	Yes	na	na	na
68	Yes	Yes	na	Yes	na	Yes	na	na	na
87	Yes	Yes	na	Yes	na	Yes	na	na	na
111	Yes	Yes	na	Yes	na	Yes	na	na	na
113	Yes	Yes	na	Yes	na	Yes	na	na	na
114	Yes	Yes	na	Yes	na	Yes	na	na	na
116	Yes	Yes	na	Yes	na	Yes	na	na	na
148	Yes	Yes	na	Yes	na	Yes	na	na	na
154	Yes	Yes	na	Yes	na	Yes	na	na	na
16	Yes	Yes	na	Yes	na	No	na	na	na
35	Yes	Yes	na	Yes	na	No	na	na	na
49	Yes	Yes	na	Yes	na	No	na	na	na
86	Yes	Yes	na	Yes	na	No	na	na	na
91	Yes	Yes	na	Yes	na	No	na	na	na

158	Yes	Yes	na	Yes	na	No	na	na	na
92	Yes	Yes	na	No	na	Yes	na	na	na
110	Yes	Yes	na	No	na	Yes	na	na	na
9	Yes	Yes	na	No	na	No	na	na	na
42	Yes	Yes	na	No	na	No	na	na	na
54	Yes	Yes	na	No	na	No	na	na	na
59	Yes	Yes	na	No	na	No	na	na	na
70	Yes	Yes	na	No	na	No	na	na	na
71	Yes	Yes	na	No	na	No	na	na	na
77	Yes	Yes	na	No	na	No	na	na	na
93	Yes	Yes	na	No	na	No	na	na	na
103	Yes	Yes	na	No	na	No	na	na	na
109	Yes	Yes	na	No	na	No	na	na	na
139	Yes	Yes	na	No	na	No	na	na	na
143	Yes	Yes	na	No	na	No	na	na	na
155	Yes	Yes	na	No	na	No	na	na	na
156	Yes	Yes	na	No	na	No	na	na	na
118	Yes	No	na	Yes	na	Yes	na	na	na
132	Yes	No	na	Yes	na	Yes	na	na	na
19	Yes	No	na	Yes	na	No	na	na	na
120	Yes	No	na	Yes	na	No	na	na	na
150	Yes	No	na	Yes	na	No	na	na	na
1	Yes	No	na	No	na	Yes	na	na	na
153	Yes	No	na	No	na	Yes	na	na	na
2	Yes	No	na	No	na	No	na	na	na
14	Yes	No	na	No	na	No	na	na	na
15	Yes	No	na	No	na	No	na	na	na
36	Yes	No	na	No	na	No	na	na	na
37	Yes	No	na	No	na	No	na	na	na
38	Yes	No	na	No	na	No	na	na	na

57	Yes	No	na	No	na	No	na	na	na
67	Yes	No	na	No	na	No	na	na	na
69	Yes	No	na	No	na	No	na	na	na
105	Yes	No	na	No	na	No	na	na	na
122	Yes	No	na	No	na	No	na	na	na
129	Yes	No	na	No	na	No	na	na	na
137	Yes	No	na	No	na	No	na	na	na
60	No	Yes	na	Yes	na	Yes	na	na	na
108	No	Yes	na	Yes	na	Yes	na	na	na
82	No	Yes	na	Yes	na	No	na	na	na
3	No	Yes	na	No	na	No	na	na	na
11	No	Yes	na	No	na	No	na	na	na
26	No	Yes	na	No	na	No	na	na	na
102	No	Yes	na	No	na	No	na	na	na
115	No	Yes	na	No	na	No	na	na	na
119	No	Yes	na	No	na	No	na	na	na
128	No	Yes	na	No	na	No	na	na	na
142	No	Yes	na	No	na	No	na	na	na
147	No	Yes	na	No	na	No	na	na	na
149	No	No	na	Yes	na	Yes	na	na	na
6	No	No	na	No	na	No	na	na	na
8	No	No	na	No	na	No	na	na	na
10	No	No	na	No	na	No	na	na	na
17	No	No	na	No	na	No	na	na	na
27	No	No	na	No	na	No	na	na	na
28	No	No	na	No	na	No	na	na	na
39	No	No	na	No	na	No	na	na	na
46	No	No	na	No	na	No	na	na	na
47	No	No	na	No	na	No	na	na	na
48	No	No	na	No	na	No	na	na	na

50	No	No	na	No	na	No	na	na	na
55	No	No	na	No	na	No	na	na	na
56	No	No	na	No	na	No	na	na	na
62	No	No	na	No	na	No	na	na	na
72	No	No	na	No	na	No	na	na	na
73	No	No	na	No	na	No	na	na	na
74	No	No	na	No	na	No	na	na	na
81	No	No	na	No	na	No	na	na	na
84	No	No	na	No	na	No	na	na	na
85	No	No	na	No	na	No	na	na	na
89	No	No	na	No	na	No	na	na	na
95	No	No	na	No	na	No	na	na	na
106	No	No	na	No	na	No	na	na	na
123	No	No	na	No	na	No	na	na	na
124	No	No	na	No	na	No	na	na	na
131	No	No	na	No	na	No	na	na	na
133	No	No	na	No	na	No	na	na	na
157	No	No	na	No	na	No	na	na	na

Cluster randomised conrolled trials (cRCTs)

29	Yes	Yes	Yes	Yes	Yes	na	Yes	Yes	Yes
152	Yes	Yes	Yes	Yes	Yes	na	Yes	Yes	No
101	Yes	Yes	Yes	Yes	Yes	na	No	Yes	Yes
151	Yes	Yes	Yes	Yes	Yes	na	No	No	No
40	Yes	Yes	Yes	Yes	No	na	No	Yes	No
162	Yes	Yes	Yes	Yes	No	na	No	No	No
78	Yes	Yes	Yes	No	Yes	na	No	Yes	No
66	Yes	Yes	No	Yes	Yes	na	Yes	Yes	Yes
31	Yes	No	Yes	Yes	Yes	na	No	Yes	No
163	Yes	No	Yes	No	Yes	na	Yes	Yes	No

5	Yes	No	No	Yes	Yes	na	Yes	No	Yes
146	Yes	No	No	Yes	Yes	na	No	Yes	Yes
53	Yes	No	No	No	No	na	No	Yes	Yes
127	Yes	No	No	No	No	na	No	Yes	Yes
112	Yes	No	No	No	No	na	No	No	Yes
22	Yes	No	No	No	No	na	No	No	No
140	No	Yes	Yes	No	No	na	No	Yes	No
88	No	Yes	Yes	No	No	na	No	No	Yes
4	No	Yes	No	No	No	na	No	Yes	Yes
104	No	Yes	No	No	No	na	No	No	Yes
45	No	Yes	No	No	No	na	No	No	No
145	No	No	Yes	No	Yes	na	No	No	Yes
79	No	No	Yes	No	No	na	No	Yes	Yes
21	No	No	Yes	No	No	na	No	No	Yes
159	No	No	Yes	No	No	na	No	No	Yes
107	No	No	No	No	Yes	na	Yes	Yes	No
44	No	No	No	No	No	na	No	Yes	No
98	No	No	No	No	No	na	No	Yes	No
20	No	No	No	No	No	na	No	No	Yes
75	No	No	No	No	No	na	No	No	Yes
80	No	No	No	No	No	na	No	No	Yes
97	No	No	No	No	No	na	No	No	Yes
7	No	No	No	No	No	na	No	No	No
12	No	No	No	No	No	na	No	No	No
41	No	No	No	No	No	na	No	No	No
94	No	No	No	No	No	na	No	No	No
100	No	No	No	No	No	na	No	No	No
130	No	No	No	No	No	na	No	No	No
134	No	No	No	No	No	na	No	No	No
141	No	No	No	No	No	na	No	No	No
